# Increase of Macrolide-Resistance in *Streptococcus pneumoniae* Strains After the Introduction of the 13-Valent Pneumococcal Conjugate Vaccine in Lima, Peru

**DOI:** 10.3389/fcimb.2022.866186

**Published:** 2022-05-09

**Authors:** Brayan E. Gonzales, Erik H. Mercado, Maria Pinedo-Bardales, Noemi Hinostroza, Francisco Campos, Eduardo Chaparro, Olguita Del Águila, María E. Castillo, Andrés Saenz, Isabel Reyes, Theresa J. Ochoa

**Affiliations:** ^1^Instituto de Medicina Tropical Alexander von Humboldt, Universidad Peruana Cayetano Heredia, Lima, Peru; ^2^Departamento de Pediatría, Hospital Nacional Docente Madre-Niño San Bartolomé, Lima, Peru; ^3^Departamento de Pediatría, Hospital Nacional Cayetano Heredia, Lima, Peru; ^4^Facultad de Medicina, Universidad Peruana Cayetano Heredia, Lima, Peru; ^5^Servicio de Pediatría de Especialidades Clínicas, Hospital Nacional Edgardo Rebagliati Martins, Lima, Peru; ^6^Oficina de Epidemiología, Instituto Nacional de Salud del Niño, Lima, Peru; ^7^Departamento de Pediatría, Hospital Nacional Daniel Alcides Carrión, Lima, Peru; ^8^Servicio de Hospitalización, Hospital de Emergencias Pediátricas, Lima, Peru

**Keywords:** *Streptococcus pneumoniae*, macrolide-resistance, invasive pneumococcal disease, pneumococcal conjugate vaccine, healthy carrier

## Abstract

*Streptococcus pneumoniae* upper respiratory infections and pneumonia are often treated with macrolides, but recently macrolide resistance is becoming an increasingly important problem. The 13-valent pneumococcal conjugate vaccine (PCV13) was introduced in the National Immunization Program of Peru in 2015. This study aimed to evaluate the temporal evolution of macrolide resistance in *S. pneumoniae* isolates collected in five cross-sectional studies conducted before and after this vaccine introduction, from 2006 to 2019 in Lima, Peru. A total of 521 and 242 *S. pneumoniae* isolates recovered from nasopharyngeal swabs from healthy carrier children < 2 years old (2 carriage studies) and samples from normally sterile body areas from pediatric patients with invasive pneumococcal disease (IPD) (3 IPD studies), respectively, were included in this study. Phenotypic macrolide resistance was detected using the Kirby-Bauer method and/or MIC test. We found a significant increase in macrolide resistance over time, from 33.5% to 50.0% in carriage studies, and from 24.8% to 37.5% and 70.8% in IPD studies. Macrolide resistance genes [*erm*(B) and *mef*(A/E)] were screened using PCR. In carriage studies, we detected a significant decrease in the frequency of *mef*(A/E) genes among macrolide-resistant *S. pneumoniae* strains (from 66.7% to 50.0%) after introduction of PCV13. The most common mechanism of macrolide-resistant among IPD strains was the presence of *erm*(B) (96.0%, 95.2% and 85.1% in the 3 IPD studies respectively). Macrolide resistance was more common in serotype 19A strains (80% and 90% among carriage and IPD strains, respectively) vs. non-serotype 19A (35.5% and 34.4% among carriage and IPD strains, respectively). In conclusion, *S. pneumoniae* macrolide resistance rates are very high among Peruvian children. Future studies are needed in order to evaluate macrolide resistance trends among pneumococcal strains, especially now after the COVID-19 pandemic, since azithromycin was vastly used as empiric treatment of COVID-19 in Peru.

## Introduction

*Streptococcus pneumoniae*, also known as pneumococcus, is the major cause of bacterial pneumonia in young children and a common cause of other infections, including sepsis, meningitis, and otitis media (O’Brien et al., 2009; Wahl et al., 2018). Approximately 300,000 deaths were caused by invasive pneumococcus worldwide in children less than 5 years old in 2015 (Dadonaite and Roser, 2018). The treatment of pneumococcal infections has become problematic due to the increase in antibiotic resistance (Cherazard et al., 2017). Moreover, it is important to consider that pneumococcal disease is preceded by asymptomatic carriage, which is especially high in children (Bogaert et al., 2004).

The prevalence of *S. pneumoniae* carriage in healthy children less than 5 years old ranges from 20.0% to 93.4% in low-income countries and is generally higher than rates reported in lower-middle income countries (Adegbola et al., 2014). Furthermore, the incidence of invasive pneumococcal disease (IPD) varies depending on several factors, including vaccine uptake, stages of life (predominant in childhood and elderly), the geographic location, and local serotype prevalence (Dion et al., 2021; Narváez et al., 2021). In general, the prevalent serotypes recovered from IPD are also frequently identified in colonized healthy children (Apte et al., 2021; Kozakova et al., 2021).

The antibiotics of the macrolide family have long been considered drugs of potential utility in the management of infections caused by *S. pneumoniae*. However, with the emergence of macrolide resistance, their clinical value in pneumococcal infections is threatened (Doern, 2006). Recent reports show that macrolide resistance in *S. pneumoniae* is geographically variable, ranging from 30 to 50% globally (Sader et al., 2018; Wang et al., 2019; Sharew et al., 2021). The main macrolide resistance mechanisms in pneumococci are ribosomal dimethylation by an enzyme encoded by *erm*(B), and efflux by an efflux pump encoded by *mef*(A), *mef*(E) or *mef*(A/E) (Cheng and Jenney, 2016; Schroeder and Stephens, 2016; Varghese et al., 2021).

More than 28 000 cases of pneumonia have been reported each year in the Peruvian population, with a fatality rate of 1.04 deaths per 100 episodes of pneumonia, and a mortality rate of 10.5 per 100 000 inhabitants among which pneumococcus remains as the main etiological agent (Ministry of Health of Peru, 2019). Pneumococcal conjugate vaccines (PCVs) are an effective intervention in reducing the incidence of disease caused by macrolide-resistant pneumococcal serotypes covered by the vaccine (Rodgers and Klugman, 2011). In Peru, PCV was introduced to the National Immunization Program as PCV7 in 2009, and was replaced by PCV10 in 2012, and PCV13 in 2015 (Davalos et al., 2016).

The aim of this study was to evaluate the variation over time of macrolide resistance in *S. pneumoniae* strains isolated as part of 2 Carriage studies in children < 2 years old and 3 IPD studies in pediatric patients < 18 years old, conducted before and after the introduction of PCV13 in Peru.

## Material and Methods

### Study Design

A temporal evaluation of the increase in macrolide resistance was conducted using data collected in five periods. We analyzed isolates of *S. pneumoniae* from patients with IPD collected during the years 2006-2008 (IPD-1) (Ochoa et al., 2010), 2009-2011 (IPD-2) (Luna-Muschi et al., 2019), and 2016-2019 (IPD-3), and isolates from healthy children (Carriage studies) collected during the years 2007-2009 (Carriage-1) (Mercado et al., 2012) and 2018-2019 (Carriage-2). The introduction of the PCV13 into the National Immunization Program in Peru (2015) was an important milestone in prevention of IPD. In this analysis, we considered studies conducted before 2015 as “pre-vaccine studies” and studies conducted after 2015 as “post-vaccine studies”.

### Studies of Invasive Pneumococcal Disease

The study population and case definition of patients with IPD from the pre-vaccine studies (IPD-1 and IPD-2) have been described by Luna-Muschi et al. (2019). In IPD-3, as in the pre-vaccine studies, we only included pediatric patients with positive cultures for *S. pneumoniae* from a normally sterile areas (blood, cerebrospinal fluid [CSF], pleural fluid, synovial fluid, or peritoneal fluid) or biopsy culture. Pediatric patients were enrolled from public hospitals and private clinical laboratories in Lima that participated in the pre-vaccine studies.

Suspected *S. pneumoniae* isolates were transported on blood agar plates (Tryptone Soy Agar with 5% sheep blood) to the Pediatric Infectious Diseases Laboratory (the central laboratory of the study) on the same day of isolation. Bacterial cultures were grown on blood agar and re-identified based on colony morphology, alpha hemolysis, Gram staining, bile solubility, and optochin susceptibility test (World Health Organization, 2003). Strains were stored in skim milk at -70°C for further analysis.

### Studies of Healthy Carrier Children

Mercado et al. (2012) described the study population for the pre-vaccine study (Carriage-1) in detail. In this analysis, we only included data from patients enrolled in public hospitals in Lima. In each study we enrolled 1,000 healthy children between 2 and 24 months of age who attended the pediatric outpatient clinics, or the vaccination clinics of five hospitals in Lima; and whose family member reported that at the time of enrollment, the child did not suffer from any important disease or infection. Although, the child could have had a mild respiratory infection (rhinorrhea, mild cough, sneezing, temperature up to 38.5°C). Nasopharyngeal swab samples were taken using Rayon^®^ (Puritan, sterile rayon tipped applicators). One sample was taken per patient. Nasopharyngeal swabs were placed in the STGG-transport-medium (skim milk, tryptone, glucose, and glycerol) and transported to the central laboratory of the study and stored at -70°C until processing. Nasopharyngeal swabs were inoculated on Todd Hewitt enrichment broth with 0.5% yeast extract for six hours at 37°C with an atmosphere of 5% CO_2_ (Vidal et al., 2011) and streaked on blood agar. Bacterial cultures and isolates were analyzed using the same conventional methods as in IPD studies. Strains were stored in skim milk at -70°C for further analysis.

### Antibiotic Susceptibility Testing

Susceptibility to macrolides were determined using erythromycin in pre-vaccine studies (IPD-1, IPD-2, and Carriage-1). In post-vaccine studies (IPD-3 and Carriage-2), we determined macrolide resistance using azithromycin. The Kirby Bauer method using antibiotic disks (Oxoid Ltd, Basingstoke, Hans, UK) for Carriage studies and the MIC test using E-test^®^ (AB Biodisk, Solna, Sweden) for IPD studies were performed on Mueller Hinton agar with 5% sheep blood and incubated for 24 hours at 37°C in an atmosphere of 5% CO_2_. Interpretation was carried out according to the Clinical and Laboratory Standards Institute (CLSI) guidelines corresponding to the existing edition of the year in which each study was carried out (**Supplementary Table S1**) (Clinical and Laboratory Standards Institute (CLSI), 2008; CLSI, 2015; CLSI, 2019).

### DNA Extraction and PCR Detection of Macrolide-Resistant Genes

DNA isolation was performed using to the Chelex extraction method reported by Walsh et al. (2011). Each pneumococcal strain was resuspended in a single tube containing 200 µl of 5% Chelex-100® solution (Bio-rad, California, United States) supplemented with 2 µl proteinase K (20 mg/ml), and incubated them for 60 minutes at 56°C. The suspension was homogenized for 10 seconds and incubated for 15 minutes at 95°C. Tubes with pneumococcal strains were homogenized and centrifuged at 13 000 rpm for five minutes and stored them at -20°C for further analysis.

Presence of macrolide resistance genes *erm*(B) and *mef*(A/E) was evaluated by conventional PCR using primers previously reported by Sutcliffe et al. (1996). Amplified products were separated in 1.5% agarose gel stained with ethidium bromide.

### Pneumococcal Serotyping

Pneumococcal isolates from IPD-1 were serotyped by Quellung reaction at a reference lab in Israel (Soroka University Medical Center, Beer-Sheva, Israel). Serotyping of *S. pneumoniae* isolates from Carriage-1 and IPD-2 was performed by Quellung reaction at the Center for Disease Control and Prevention (CDC), Atlanta, USA. Isolates from post-vaccine studies (Carriage-2 and IPD-3) were serotyped by while genomic sequencing (WGS) in the reference laboratory, CDC Streptococcus Laboratory, USA.

### Statistical Analysis

Statistical analyses of frequency and comparison between the populations of IPD and Carriage studies were performed with chi-square test (χ2) and Fisher’s exact test in the statistical package Stata/SE v.17.0 program. The statistical significance level was set at p < 0.05.

### Ethical Aspects

This study, as well as all studies included in our analysis, were approved by the Institutional Review Board of Universidad Peruana Cayetano Heredia (Lima, Peru).

## Results

### Macrolide Resistance Phenotype

**Figure 1:**
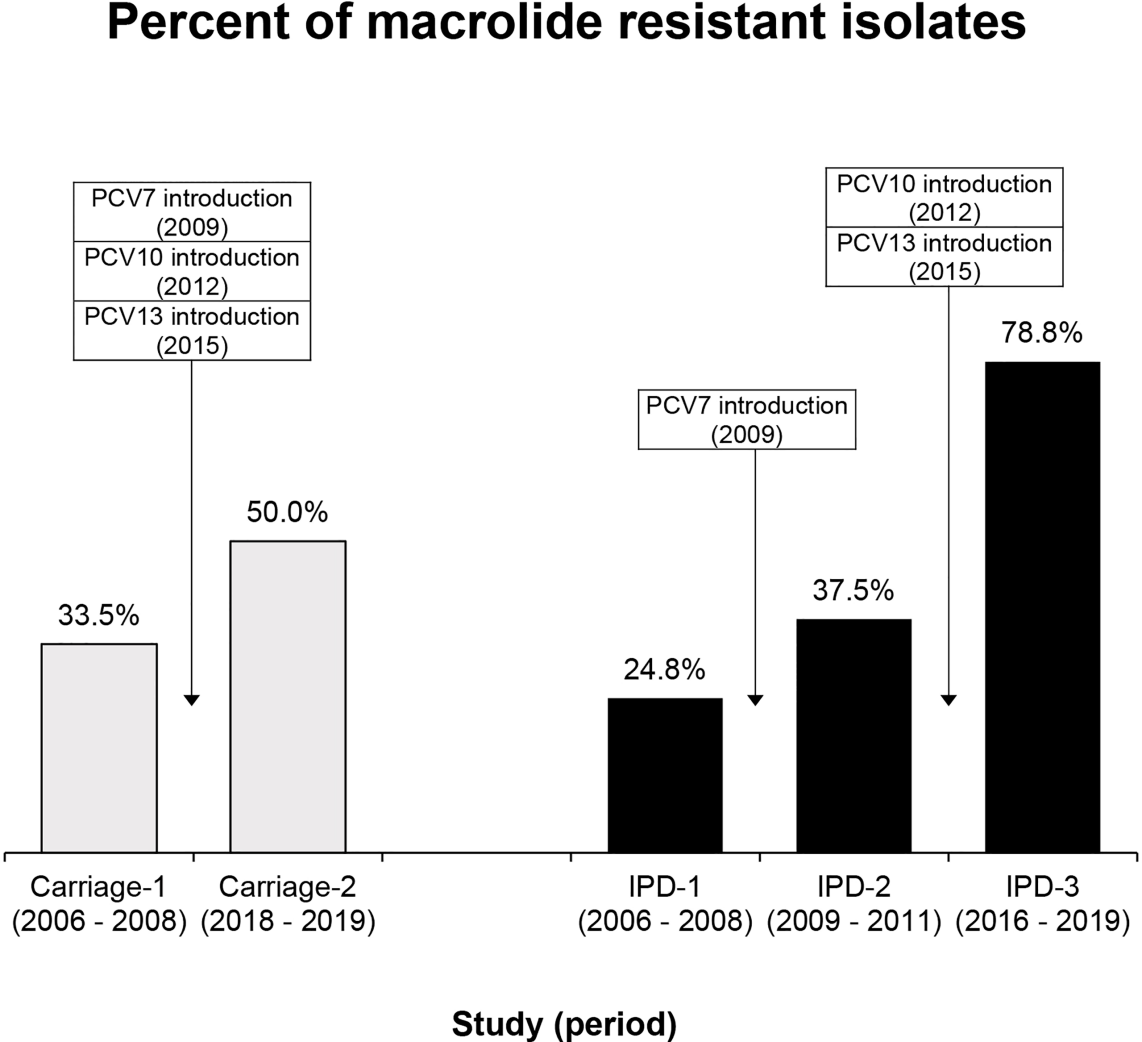
Proportion of macrolide resistance in *S. pneumoniae* isolates in Carriage and IPD studies conducted from 2006 to 2019 in Peru. Carriage-1 (105/313), Carriage-2 (104/208), IPD-1 (25/101), IPD-2 (21/56) and IPD-3 (67/85). Statistical significance among carriage and among IPD studies is p<0.001.

A total of 763 *S. pneumoniae* isolates were analyzed from the Carriage (521) and the IPD (242) studies. A significant increase in the macrolide resistance phenotype was found over time (**Table 1** and **Figure 1**). Among carriage studies, the percentage of macrolide resistance increased significantly from 33.5% in the pre-vaccine study to 50.0% in the post-vaccine study (p < 0.001). Furthermore, in IPD studies, the percentages of macrolide resistance increased significantly from 24.8% (IPD-1) and 37.5% (IPD-2) to 78.8% in IPD-3 (p < 0.001). Global macrolide resistance in pre-vaccine studies (including IPD-1 and IPD-2 pneumococcal isolates) was 29.3% (46/157).

**Table 1:** Macrolide susceptibility in 5 studies conducted from 2006 to 2019 before and after the introduction of PCV13 in Peru.

Study	Study Period	Susceptibility		Number of strains	S		I		R		p
		Method	Antibiotic		n	(%)	n	(%)	n	(%)	
Carriage-1^a^	2006-2008	Disk diffusion	Erythromycin	313	195	(62.3)	13	(4.2)	105	(33.5)	< 0.001
Carriage-2^b^	2018-2019	Disk diffusion	Azithromycin	208	88	(42.3)	16	(7.7)	104	(50.0)	
IPD-1^a^	2006-2008	MIC	Erythromycin	101	76	(75.2)	0	(0.0)	25	(24.8)	< 0.001
IPD-2^a^	2009-2011	MIC	Erythromycin	56	34	(60.7)	1	(1.8)	21	(37.5)	
IPD-3^b^	2016-2019	MIC	Azithromycin	85	12	(14.1)	6	(7.1)	67	(78.8)	

13-valent conjugate vaccine (PCV13) was inserted into the National Immunization Program in 2015.

a Pre-vaccine studies (Pre PCV13).

b Post-vaccine studies (Post PCV13).

S, susceptible; I, intermediate; R, resistant.

Statistical significance among carriage studies and among IPD studies.

### Macrolide Resistance Genes

Among *S. pneumoniae* strains resistant to macrolides from carriage studies, a total of 80% (84/105) of the strains from the pre-vaccine period and 100% (104/104) of the strains from the post-vaccine period were analyzed to determine the presence of macrolides resistance genes, *erm*(B) and *mef*(A/E). 21 strains from the Carriage pre-vaccine period were not recovered.

In Carriage studies, the most common macrolide resistance gene was *mef*(A/E), and its prevalence was significantly lower in the post-vaccine period than in the pre-vaccine period (**Table 2**). In Carriage post-vaccine study, *erm*(B) and *mef*(A/E) were present with similar frequency. There were 2 macrolide-resistant isolates without any of the evaluated genes in pre- and post-vaccine studies, respectively.

**Table 2:** Distribution of macrolide resistance genes.

Study		*erm*(B)		*mef*(A/E)		only *erm*(B)		only *mef*(A/E)		*erm*(B) + *mef*(A/E)	
	N	Positive	p	Positive	p	Positive	p	Positive	p	Positive	p
		n (%)		n (%)		n (%)		n (%)		n (%)	
Healthy carrier children’s			1.000		0.022		0.953		0.026		0.892
Carriage-1^a^	84	42 (50.0)		56 (66.7)		26 (30.9)		40 (47.6)		16 (19.1)	
Carriage-2^b^	104	52 (50.0)		52 (50.0)		33 (31.7)		33 (31.7)		19 (18.3)	
Invasive pneumococcal disease			0.296		0.070		0.103		0.435		0.134
IPD-1^a^	25	24 (96.0)		12 (48.0)		12 (48.0)		0 (0.0)		12 (48.0)	
IPD-2^a^	21	20 (95.2)		17 (80.9)		4 (19.1)		1 (4.8)		16 (76.2)	
IPD-3^b^	67	57 (85.1)		42 (62.7)		20 (29.8)		5 (7.5)		37 (55.2)	

13-valent conjugate vaccine (PCV13) was inserted into the National Immunization Program in 2015.

a Pre-vaccine studies (Pre PCV13).

b Post-vaccine studies (Post PCV13).

Statistical significance among carriage studies and among IPD studies.

The *erm*(B) gene represents the most common mechanism of macrolide resistance among IPD strains. This gene was detected in 96.0% of isolates from IPD-1, 95.2% in IPD-2, and 85.1% in IPD-3 (not statistically different) (**Table 2**). None of these genes were detected in one and five macrolide-resistant isolates from IPD-1 and IPD-3 respectively.

### Macrolide Resistance of 19A Serotype Pneumococcal Isolates

We were able to serotype 472/521 pneumococcal isolates from Carriage and 236/242 pneumococcal isolates from IPD studies. **Table 3** shows the 5 most prevalent serotypes from each study, with macrolide resistance rates and resistance genes. We found that the 19A serotype was significantly associated with macrolide resistance in comparison with non-19A serotypes, 80.0% vs. 35.5%, respectively, among carriage strains, and 90.0% vs. 34.4%, respectively, among IPD strains. Macrolide-resistant 19A isolates from carriers were associated with the presence of the *erm*(B) gene (p=0.002), while macrolide-resistant 19A isolates from IPD patients were associated with *mef*(A/E) genes (p<0.001).

**Table 3:** Most prevalent pneumococcal serotypes, macrolide resistance and genes in carriage and IPD.

Most prevalent serotypes			Susceptibility		*erm*(B)	*mef*(A/E)
	n (%)	N	Resistant	N	Positive	Positive
			n (%)		n (%)	n (%)
Carriage-1 (N=280)^a^						
19F	59 (21.1)	59	30 (50.9)	30	17 (56.7)	28 (93.3)
6B	33 (11.8)	33	12 (36.4)	12	10 (83.3)	2 (16.7)
23F	24 (8.6)	24	7 (29.2)	7	2 (28.6)	5 (71.4)
14	13 (4.6)	13	0	–	–	–
19A^d^	9 (3.2)	9	4 (44.4)	4	4 (100.0)	0
Carriage-2 (N=208)^c^						
15C	24 (11.5)	24	9 (37.5)	9	1 (11.1)	7 (77.8)
19A	21 (10.1)	21	20 (95.2)	20	15 (75.0)	11 (55.0)
6C	21 (10.1)	21	15 (71.4)	15	3 (20.0)	12 (80.0)
23A	17 (8.2)	17	16 (94.1)	16	11 (68.8)	3 (18.8)
15B	15 (7.2)	15	0	–	–	–
IPD-1 (N=99)^a^						
14	26 (26.3)	26	3 (11.5)	3	3 (100.0)	2 (66.7)
6B	20 (20.2)	20	8 (40.0)	8	8 (100.0)	0
19F	11 (11.1)	11	7 (63.6)	7	7 (100.0)	6 (85.7)
23F	6 (6.1)	6	1 (16.7)	1	0	0
19A^e^	4 (4.0)	4	3 (75.0)	3	3 (100.0)	2 (66.7)
IPD-2 (N=58)^b^						
14	11 (19.0)	11	3 (27.3)	3	2 (66.7)	3 (100.0)
19F	9 (15.6)	9	8 (88.9)	8	8 (100.0)	8 (100.0)
23F	6 (10.3)	6	4 (66.7)	4	4 (100.0)	4 (100.0)
6B	6 (10.3)	6	3 (50.0)	3	3 (100.0)	0
19A	5 (8.6)	5	1 (25.0)	1	1 (100.0)	1 (100.0)
IPD-3 (N=81)^c^						
19A	42 (51.9)	42	41 (97.6)	41	36 (87.8)	38 (92.7)
24F	19 (23.5)	19	18 (94.7)	18	15 (83.3)	0
16F	2 (2.5)	2	0	–	–	–
23B	2 (2.5)	2	1 (50.0)	1	1 (100.0)	1 (100.0)
35B	2 (2.5)	2	0	–	–	–

13-valent conjugate vaccine (PCV13) was inserted into the National Immunization Program in 2015.

a Pre-PCV7 introduction.

b Post-PCV7 introduction.

c Post-PCV13 introduction.

d Serotype 19A was number 9 among the most prevalente serotypes in Carriage-1.

e Serotype 19A was number 6 among the most prevalente serotypes in IPD-1.

## Discussion

Our results show a significant increase in macrolide resistance through time in *S. pneumoniae* strains isolated in 2 Carriage studies in healthy carrier children, and 3 IPD studies in ill pediatric patients, conducted before and after the introduction of PCV13.

In the post-vaccine Carriage study, 50.0% of pneumococcal isolates were macrolide-resistant. A nasopharyngeal carriage study conducted in Cajamarca, an Andean region of Peru, reported that the frequency of erythromycin-resistant isolates before the introduction of PCV7 was 13.6% (among 125 pneumococcal strains in children < 3 years old) and 22.4% (among 125 pneumococcal strains in children < 3 years old) after the introduction of PCV7, a lower prevalence than our study (Hanke et al., 2016). In this vaccination period, the frequency of macrolide resistance was evaluated in 4 surveillance studies in Greece; they reported no significant change through time: 22.0% in 2005; 33.3% in 2006; 23.7% in 2007; and 20.5% in 2009 (Grivea et al., 2012). Furthermore, our prevalence is higher than the one reported in Cyprus after the introduction of PCV10 and PCV13, where 20.7% of all *S. pneumoniae* isolates were erythromycin-resistant (Hadjipanayis et al., 2016). A similar study conducted in carrier children Although our results show a significant increase, it is lower than the reported by Hashida et al. (2011) before PCV7 was introduced in the Japan’s routine immunization program, with almost 69.4% macrolide-resistant pneumococcal isolates from children who attended daycare centers.

In IPD studies, the percentage of macrolide-resistant strains increased significantly and progressively: 24.8% in IPD-1, 37.5% in IPD-2, and 78.8% in IPD-3. There no other IPD studies in Peru to compare with our data. However, in other countries the results are different. For example, in the United States, the proportion of erythromycin-resistant isolates have not changed significantly after the introduction of PCV7: 19.4% in 1999, and 18.1% in 2004 (Kyaw et al., 2006). This prevalence is similar to the one described by Farrell *et al*. from American pediatric patients (< 14 years old) with IPD between 2000 and 2004. Additionally, they observed a decrease in the cases of IPD caused by vaccine serotypes and an increase in the cases of IPD by non-vaccine serotypes. In this study, the macrolide resistance did not change (from 40.6% to 38.7%), but the rate of resistance to macrolides in the non-vaccine serotypes increased from 21.0% to 27.0% (Farrell et al., 2007). In Brazil, before and after introduction of PCV10 the frequency of macrolide-resistant isolates was 12.0% and 21.0% respectively (Almeida et al., 2021). In Germany, the frequency of macrolide-resistant pneumococcus after the introduction of PCV7 decreased from 24.7% to 17.2%, and it continued decreasing after the introduction of PCV13 (8.2%) (Imöhl et al., 2015). However, it has been observed that the frequencies of macrolide-resistant *S. pneumoniae* from IPD patients changed temporally and geographically, before and after the introduction of PCVs. Probably the reason why macrolide resistance has not decreased in Peru after the introduction of PCVs is because some resistant serotypes, such as 19A, are still very common, especially among invasive diseases, mainly due to recent introduction of PCV13.

On the other hand, a current meta-analysis, conducted by Andrejko et al. in 2021, evaluated the impact of the introduction of PCVs on antibiotic resistance in more than 300,000 pneumococcal strains worldwide, isolated from carriers and IPDs, but they did not find a significant change in the proportion of non-susceptible isolates after the introduction of PCVs (Andrejko et al., 2021). This is different from our study, where we reported an increase in macrolide resistance in both groups.

In Carriage studies, *mef*(A/E) was the most common gene, and its frequency decreased significantly from 66.7% to 50.0% after the introduction of PCV13. This frequency was comparable with the proportion found in Greece after the introduction of PCV7, where *mef*(A/E) genes were detected in 47.1% of total macrolide-resistant isolates from 2005 to 2009. Even though, in that country, unlike in Peru, the frequency of these genes increased after the introduction of PCV7 in the National Immunization Plan (41.6% one year before the introduction and 78.0% four years after the introduction) (Grivea et al., 2012). Other studies showed a decrease in the frequency of *mef*(A/E) in macrolide-resistant *S. pneumoniae* isolates in post-vaccine studies in countries with low immunization coverage, such as Russia, where PCV7 was marketed in 2009 but has never been offered for mass vaccination. In 2006, before the introduction of PCV7 in Russia, *mef*(A) was found in 43.8% of pneumococcal isolates (Vorobieva S Jensen et al., 2020), but after PCV7 the frequency of *mef* genes decreased to 13.0% (Mayanskiy et al., 2014). The decrease in the prevalence of *mef*(A/E) in *S. pneumoniae* from nasopharyngeal carrier children could be affected by other factors different than the introduction of PCVs.

In the post-vaccine Carriage study, *erm*(B) gene and, *erm*(B) + *mef*(A/E) were detected in 50.0% and 18.3%, respectively, but a statistically significant association was not found. In Jordan, after the introduction of PCV, *erm*(B) and [*erm*(B) + *mef*(A/E)] genes were detected in 50.0%, and 1.7% of macrolide-resistant *S. pneumoniae* (Al-Lahham et al., 2018). Furthermore, post-introduction of PCV7 in Jordan, Swedan et al. (2016) found that 43.8% of pneumococcal isolates in carrier children under 5 years, presented *erm*(B), and 6.3% both genes. Even though the *erm*(B) + *mef*(A/E) prevalence is lower, the *erm*(B) frequencies are similar to ours, despite the fact that these studies were conducted in years of low vaccination rates in Jordan.

In pre and post vaccine IPD studies, *erm*(B) was the most frequent gene (85.0% - 96.0%) in macrolide-resistant isolates but, as well *mef*(A/E), the presence of this gene was not found significantly associated with the introduction of PCVs. Our *erm*(B) frequency was similar that reported in Colombia before PCV13 (98.0%) (Ramos et al., 2014). On the other hand, in Brazil before the introduction of PCV13 *erm*(B) was detected in an lower frequency, 10.0% of intermediate or resistant erythromycin *S. pneumoniae* isolates and 7.0% of those isolates had both genes (Almeida et al., 2021). In the United States, after the introduction of PCV7, was found an increase in 18.0% the proportion of strains positive for both genes between the first and last year of surveillance study, with a corresponding decrease in the prevalence of positive isolates for only *mef*(A) (72.0% to 58.0%) and only *erm*(B) (15.8% to 11.2%) (Farrell et al., 2007). In Germany the *mef*(A) gene decreased in the post vaccine period (13.3% to 3.9% to 0.6%) but the presence of the *erm*(B) gene remained stable (between 3.9% and 5.4%); in addition, an increase in the prevalence of isolates with both genes *mef*(A) and *erm*(B) (0.0% to 0.8% to 1.7%) was observed (Imöhl et al., 2015). On the other hand, in Asia, their antibiotic resistance surveillance system reported that after PCV7 introduction, the macrolide-resistant pneumococcal isolates from IPD patients presented different genotype frequencies among the countries evaluated (Kim et al., 2012). This information support that the distribution of genotypes among macrolide-resistant *S. pneumoniae* isolates is specific to each country and it is difficult to predict (Kim et al., 2012). We recommend the implementation of a specific surveillance plan to monitor the circulation of drug-resistant pathogens in each country and antimicrobial use.

Before the introduction of PCV13, *mef*(A/E)-encoded efflux pumps were the most common mechanism of macrolide resistance in North America, the United Kingdom, and some European countries (Schroeder and Stephens, 2016). However, in most countries in Europe, Asia and Latin America, *erm*(B) remained the most frequent mechanism. In 2012, the dual macrolide resistance genotype was reported with frequencies up to 52% (Bowers et al., 2012). Among isolates with this dual genotype, *Tn2010* was identified as the main mobile element containing *erm*(B) and *mef*(E)/*mel* (Del Grosso et al., 2007). Due to the pressure exerted by the first PCV (PCV7), the *Tn2010* “replacement” serotype 19A (ST320) emerged (Del Grosso et al., 2007; Schroeder and Stephens, 2016). ST320 is a multi-drug resistant strain that represents a “capsule switch” from serotype 19F; 19A (ST320) quickly became one of the most prevalent serotypes (Moore et al., 2008; Schroeder and Stephens, 2016; Agudelo et al., 2018). We believe that this replacement is related to the decreased presence of *mef*(A/E) genes and increased macrolide resistance. Further studies by WGS are need in our strains in order to confirm the presence of ST320 strains in Peru.

Serotype 19A was the predominant serotype in our IPD post-vaccine study and the second most common in healthy carrier children, the most common serotype was 15C (Abstract submitted to ISPPD 2022), and in both cases was associated with macrolide resistance. Additionally, the presence of only *erm*(B) and the presence of both genes were associated with this serotype (79.2% and 37.5%, respectively). In Jordan, a study in healthy carrier children reported that among serotype 19A isolates, 50.0% had *erm*(B) gene, 44.9% *mef*(A) and 1.7% both genes, in all cases, lower than the frequencies reported by us (Al-Lahham et al., 2018). In Spain, 71.5% of the serotype 19A strains presented resistance to erythromycin, similar to our findings (Alfayate Miguélez et al., 2020). However, there is still not enough evidence on the distribution of serotypes and their resistance patterns in isolates of pneumococcal carriage children.

*S. pneumoniae* serotype 19A has emerged as one of the leading causes of invasive disease in many countries, regardless of whether conjugate pneumococcal vaccines are used (Isturiz et al., 2010). Among serotype 19A pneumococcal isolates from patients with IPD, 90.0% were resistant to macrolides (**Table 4**), higher than proportion detected in the United States (45.5%) (Farrell et al., 2007), and the average frequency in countries from Latin America (Bolivia, Brasil, Chile, Costa Rica, Republica Dominicana, Ecuador, El Salvador, Guatemala, Nicaragua, Panama, Paraguay y Uruguay) (43.3%) (Moreno et al., 2020). Additionally, our study reports that the presence of only *mef*(A/E) and both genes [*erm*(B) + *mef*(A/E)], was associated with serotype 19A (91.1% and 84.4% respectively). In Brazil, 44.0% of pneumococcal carriers presented the *mef*(A) gene, 36.0% *erm*(B) and both 20.0%, in contrast to our study, serotype 19A was not associated with erythromycin resistance (Caierão et al., 2014). In 2014, the results of the System of Surveillance Networks for Agents Responsible for Bacterial Pneumonia and Meningitis (SIREVA) revealed that serotype 19A prevalence varies from 0% in Nicaragua, Guatemala, and El Salvador to 36.7% in Peru (Pan American Health Organization, 2017). Due to the high prevalence of resistance to macrolides in this serotype, active surveillance is needed to evaluate its temporal and geographical evolution, which will allow better public health actions to control the spread of this serotype among healthy carrier children, who are vulnerable to developing pneumococcal disease (Løvlie et al., 2020).

**Table 4:** Macrolide resistance and genes among 19A pneumococcal isolates in carriage and IPD strains.

Serotype		Susceptibility			*erm*(B)		*mef*(A/E)		*erm*(B) + *mef*(A/E)	
	N	Resistant	p	N	Positive	p	Positive	p	Positive	p
		n (%)			n (%)		n (%)		n (%)	
Carriage studies			< 0.001			0.002		0.163		0.012
19A	30	24 (80.0)		24	19 (79.2)		11 (45.8)		9 (37.5)	
Another serotype	442	157 (35.5)		156	71 (45.5)		95 (60.9)		25 (16.0)	
IPD studies			< 0.001			0.767		< 0.001		< 0.001
19A	50	45 (90.0)		45	40 (88.9)		41 (91.1)		38 (84.4)	
Another serotype	186	64 (34.4)		64	58 (90.6)		28 (43.8)		26 (40.6)	

Statistical significance among carriage studies and among IPD studies.

Our study had some limitations. The focus of our study was on the most common macrolide-resistance mechanisms and we did not test the presence of the less common macrolide-resistance mechanisms, such as *erm* (A), *erm* (TR), *mef* (I), *msr* (D), *rpID* and *rpIV* (Schroeder and Stephens, 2016*)*. Also, we used different methods of antibiotic susceptibility testing in the pre and post vaccine studies. Additional studies are needed to elucidate the potential impact of PCVs on the distribution of *mef*(A/E). Despite these limitations, this is the first report of the increase in macrolide-resistance in *Streptococcus pneumoniae* strains after the introduction of the PCV13 vaccine in Lima, Peru. We strongly recommend a new study to evaluate the frequency of macrolide resistance among pneumococcal strains after the COVID-19 pandemic, since this family of antibiotics, especially azithromycin, was widely used for empiric treatment against SARS-CoV-2 in Peru, which could greatly affect the prevalence and mechanisms of macrolide resistance.

## Data Availability Statement

The raw data supporting the conclusions of this article will be made available by the authors, without undue reservation.

## Ethics Statement

The studies involving human participants were reviewed and approved by Comité Institucional de Ética en Investigación (CIEI) - Humanos Universidad Peruana Cayetano Heredia. Written informed consent to participate in this study was provided by the participants’ legal guardian/next of kin.

## Author Contributions

BG and EM initiated and designed the macrolide research study. BG and MP-B wrote the first draft of the manuscript. BG, MP-B and NH performed the experiments (antibiotic susceptibility and PCRs) and analyzed and/or interpreted the results. FC, EC, OÁ, MC, AS, IR and TO were in charge of the design and conduct of all five surveillance studies, patient enrollment, sample collection and data acquisition. All authors contributed to the article and approved the submitted version.

## Funding

The Carriage-1 study was funded by an institutional grant from of “Alberto Hurtado” School of Medicine at Universidad Peruana Cayetano Heredia, awarded to Dr. Theresa Ochoa. The Carriage-2 study was funded by internal funds of the Pediatric Infectious Diseases Laboratory at the Universidad Peruana Cayetano Heredia. Finally, all IPD studies were supported by a research grant from Pfizer Laboratories to Grupo Peruano de Investigación en Neumococo (GPIN, Peruvian research group on pneumococcus). The external funders had no role in study design, data collection and interpretation, or the decision to submit the work for publication.

## Conflict of Interest

The authors declare that the research was conducted in the absence of any commercial or financial relationships that could be construed as a potential conflict of interest.

## Publisher’s Note

All claims expressed in this article are solely those of the authors and do not necessarily represent those of their affiliated organizations, or those of the publisher, the editors and the reviewers. Any product that may be evaluated in this article, or claim that may be made by its manufacturer, is not guaranteed or endorsed by the publisher.
