# Supplementary material for: Increase of Macrolide-Resistance in Streptococcus pneumoniae Strains After the Introduction of the 13-Valent Pneumococcal Conjugate Vaccine in Lima, Peru
Source: Front Cell Infect Microbiol. 2022 May 9;12:866186. doi: 10.3389/fcimb.2022.866186 (PMC9125093; doi:10.3389/fcimb.2022.866186)
Supplement: Supplementary file 1 [file Table_1.docx]

| **Method** | **Antibiotic** |  | **Disk content** |  | **Zone diameter, nearest whole mm** | | |
| --- | --- | --- | --- | --- | --- | --- | --- |
|  |  |  |  |  | **S** | **I** | **R** |
| Disk diffusion | Erythromycin |  | 15 µg |  | > 21 | 16-20 | < 15 |
|  | Azithromycin |  | 15 µg |  | > 18 | 14-17 | < 13 |
|  |  |  |  |  |  |  |  |
|  |  |  | **MIC range** |  | **Equivalent MIC breakpoints (µg/mL)** | | |
|  |  |  |  |  | **S** | **I** | **R** |
| MIC | Erythromycin |  | 0.016-256 µg/mL |  | < 0.25 | 0.5 ^a^ | > 1 |
|  | Azithromycin |  | 0.016-256 µg/mL |  | < 0.5 | 1 | > 2 |

Supplementary Material

**Supplementary Table S1.** Macrolide susceptibility breakpoints.

S, susceptible; I, intermediate; R, resistant.

CLSI 2008 guidelines were used in Carriage-1 and IPD-1.

CLSI 2015 guidelines were used in IPD-2.

CLSI 2019 guidelines were use in Carriage-2 and IPD-3.

^a^CLSI 2008 did not include breakpoints to intermediate category.
